# Liquid immersion thermal crosslinking of 3D polymer nanopatterns for direct carbonisation with high structural integrity

**DOI:** 10.1038/srep18185

**Published:** 2015-12-18

**Authors:** Da-Young Kang, Cheolho Kim, Gyurim Park, Jun Hyuk Moon

**Affiliations:** 1 Department of Chemical and Biomolecular Engineering, Sogang University, Seoul 121-742, Republic of Korea

## Abstract

The direct pyrolytic carbonisation of polymer patterns has attracted interest for its use in obtaining carbon materials. In the case of carbonisation of nanopatterned polymers, the polymer flow and subsequent pattern change may occur in order to relieve their high surface energies. Here, we demonstrated that liquid immersion thermal crosslinking of polymer nanopatterns effectively enhanced the thermal resistance and maintained the structure integrity during the heat treatment. We employed the liquid immersion thermal crosslinking for 3D porous SU8 photoresist nanopatterns and successfully converted them to carbon nanopatterns while maintaining their porous features. The thermal crosslinking reaction and carbonisation of SU8 nanopatterns were characterised. The micro-crystallinity of the SU8-derived carbon nanopatterns was also characterised. The liquid immersion heat treatment can be extended to the carbonisation of various polymer or photoresist nanopatterns and also provide a facile way to control the surface energy of polymer nanopatterns for various purposes, for example, to block copolymer or surfactant self-assemblies.

The direct carbonisation of polymer materials has been widely demonstrated to produce carbon materials. The beauty of this approach is that carbon with various morphologies can be obtained using a variety of polymer materials or patterns (e.g., block copolymer assemblies, patterns, and polymer spheres) as a precursor^1^,^2^,^3^,^4^,^5^,^6^,^7^. Moreover, compared to using soft or hard templates for the fabrication of porous carbon, the direct carbonisation is more simple and facile^8^,^9^. Specifically, the carbonisation of photoresist polymer patterns allows the lithography-based design of carbon materials, one of the well-established and highly delicate microfabrication techniques^3^,^5^,^10^,^11^,^12^,^13^. The fabrication of micropatterned carbons has been successfully demonstrated and has led to new applications of carbon materials in MEMS, electrochemical sensors and energy devices^3^,^10^,^11^,^12^,^13^,^14^.

Polymer carbonisation is typically processed at high temperatures (usually above 500 °C) to induce pyrolytic reduction of the polymers into carbonaceous materials^2^. This high-temperature treatment of polymer patterns may result in problems that need to be addressed carefully. (1) First, the polymer may flow (e.g., at the temperature above the glass transition temperature (*T*_*g*_) of the polymer) and the pattern morphology can be changed to lower the surface energy, which makes it difficult to obtain the desired carbon patterns. (2) Moreover, a large mass loss during carbonisation at high temperature may also lead shrinkage of the macroscopic morphology of the produced carbon. Previously, these macroscopic morphology changes were reported less frequently. This may be attributed to mostly use the template materials for the fabrication of porous structures. The templates maintains the structure during the carbonisation^8^,^9^. Moreover, most of the lithography-patterned polymeric patterns used for direct carbonisation were thin and film-like^3^,^5^,^6^,^11^,^12^,^15^. In this case, strong adhesion with the substrate may resist macroscopic pattern change during carbonisation. However, an attempt to utilise polymer patterns with high-aspect-ratio or high-specific-area patterns (e.g., 3D or porous patterned film) in direct carbonisation may be more likely to encounter this problem. For example, as shown in Figure S1, we prepared a photoresist polymer pattern with a 3D pore network and submicrometer features using a lithography technique and investigated the morphology during the heat treatment. We found that the polymer patterns flowed and then induced pore collapse at only 150 °C (much lower than the carbonisation temperature), resulting in pattern-collapsed carbon films. Thus, a method to improve the thermal resistivity of polymer patterns may first be required to maintain the structural integrity during the direct carbonisation.

Here, we utilized the thermal crosslinking of polymer patterns. Crosslinking can retard polymer flow by weakening the glass transition of the polymer and the subsequent macroscopic polymer flow^16^. In addition, crosslinking of polymer patterns may enhance carbon conversion by producing less volatile moieties during the pyrolytic carbonisation^17^. However, this thermal crosslinking may induce the same problem of pattern flow and subsequent collapse, as shown above. Thus, in this paper, we describe a novel approach for the thermal crosslinking of polymer nanopatterns while retaining the structural integrity and successful carbon conversion. We found that the thermal crosslinking by immersing in liquid solvent was effective at relieving the high surface energy of the nanopatterns and thereby maintain the pattern morphology during the heat treatment. We also observed that the crosslinked nanopatterns maintained their pattern or pore structure during direct pyrolytic carbonisation, along with large mass loss. We believe the utility of this approach is not limited to the SU8 photoresist; it could also be applied to a variety of polymers or photoresists that can be thermally crosslinked. Moreover, this approach can provide a facile way to control the interfacial energy of polymer nanopatterns during heat treatment for various purposes, for example, thermally induced block copolymer self-assemblies and hard-baking of polymer patterns.

## Experimental

### Multi-beam interference lithography for 3D nanopattern generation

A 3D porous SU8 nanopattern was obtained via five-beam interference lithography. The SU8 photoresist was prepared by dissolving 10 wt% of SU-8 (Miller-Stephenson) in a γ–butyrolactone (Sigma-Aldrich) and then, adding a cationic photoinitiator, (5-Cyclopentadienyl) (6-isopropylbenzene) iron hexafluorophosphate by 2 wt% (over the SU8). The photoresist solution was spin-coated and subsequently baked at 95 °C, resulting in the photoresist film with a thickness of 7–9 μm. Meanwhile, the interference pattern for wood-pile symmetry 3D nanopatterns was generated by passing the laser (Nd:YVO_4_, 532 nm, 20 mJ/cm^2^, Coherent Inc.) beam through a top-cut four-sided prism. The beam expanded was located before the exposure onto the prism. The beam directed onto the top surface and the four beams directed to the prism sides possess the wavevectors of *k*_*0*_ = *k*(0, 0, 0), *k*_*1*_ = *k* (–0.36, 0, 0.93), *k*_*2*_ = *k* (0, –0.36, 0.93), *k*_*3*_ = *k* (0.36, 0, 0.93), and *k*_*4*_ = *k* (0, 0.36, 0.93), respectively, where *k* = 2π/*λ*, *λ* is the wavelength of the laser beam (532 nm). We placed the prism on the photoresist film and conducted the exposure. The post-exposure baking was followed at 65 °C for 5 min and 95 °C for 1 min. The 3D nanopatterns were developed by soaking in a propylene glycol methyl ether acetate (Sigma-Aldrich) solution for around 5 mins.

### Characterisation

The surface morphologies were measured by a field emission scanning electron microscope (FESEM, Hitachi, S-4700). X-ray photoelectron spectroscopy (XPS, Thermo Fisher Scientific, ESCALAB 250 XPS System) analysis using a monochromated Al Ka x-ray source (hv = 1486.6 eV) at a chamber pressure of 1 × 10–10 torr was performed for elemental analysis of the nanopattern carbon. Raman spectra were recorded using micro Raman spectroscopy (Tokyo instrument, Nanofinder) with an excitation wavelength of 487.55 nm. Thermogravimetric analysis (TGA) was measured by heating the sample up to 900 °C in a nitrogen atmosphere, with a heating rate of 4 °C/min (TA instrument TGA Q50). The contact angle of liquid solvent on the SU8 film was measured with a contact angle goniometer (Phoenix 300, Surface Electro Optics). The FT-IR spectrum were measured by using Nicolet FT-IR spectrometer. Nanoindentation was conducted using an MTS XP (MTS systems corporation). The indenter tip (Berkovich type triangular pyramid) was loaded and run in a depth-controlled mode.

## Results and Discussion

### Fabrication of 3D porous nanopatterns and liquid immersion thermal crosslinking

**Figure 1 f1:**
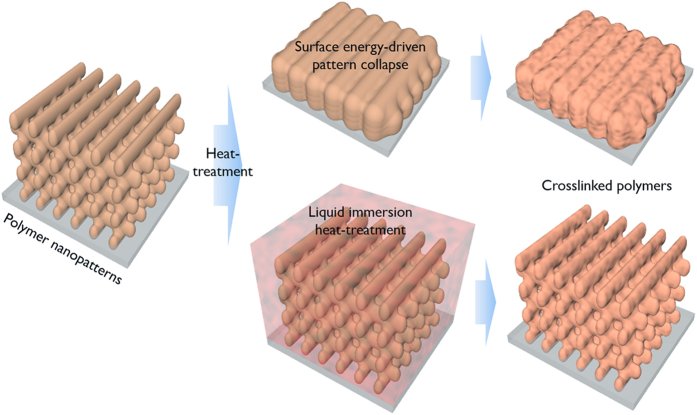
Comparative scheme of liquid-immersion heat-treatment for the thermal crosslinking of polymer nanopatterns.

**Figure 2:**
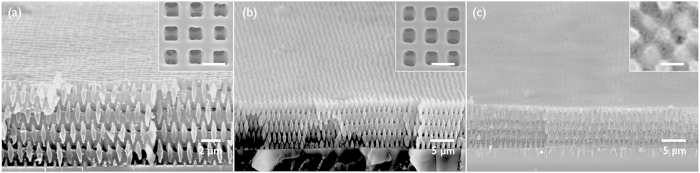
(**a**) SEM image of SU8 wood-pile patterns in a tilted view. (**b**,**c**) SEM images of wood-pile patterns treated at 200 °C immersed in hexadecane and triethylene glycol, respectively. The insets show the surface image of each pattern. (Scale bar, 1 μm).

A 3D porous nanopattern was obtained via multi-beam laser interference lithography using commercial SU8 photoresist, as described in Figure S1. This pattern was utilised as a model pattern with submicrometer and porous features; however, the carbon patterns can be used in unique applications such as electrochemical electrodes, sensors and MEMS devices^5^,^11^,^12^. Upon exposure to laser interference and subsequent *post-exposure baking*, the proton released from the initiator in the SU8 photoresist polymerised and crosslinked the epoxide groups in the SU8 molecules. The post-exposure baking of the film was typically achieved below 100 °C. Heat treatment at higher temperature induced too much initiator diffusion, resulting in low pattern contrast^18^. The crosslinked SU8 region was left behind in the developer solution.[Fig f1] Figure 2a exhibits the 3D porous nanopatterns of the SU8. The pattern possessed a wood-pile-like symmetry with a 500 nm line pattern width, 950 nm line-to-line distance, and 2 μm layer-by-layer distance. The patterned SU8 polymers typically display a low crosslinking degree (below 10%) and possess a *T*_*g*_ similar to the temperature used for the post-exposure baking^19^. Thus, further crosslinking of SU8 polymer nanopatterns can be achieved via the unreacted epoxide groups. However, as aforementioned, we discovered that direct thermal crosslinking induced pattern collapse. The nanopattern lost its porous morphology (i.e., the surface area decreased) to relieve its surface energy. Our approach was intended to lower the surface energy of the photoresist nanopatterns during thermal crosslinking. Here, thermal crosslinking was achieved while the nanopattern was immersed in a liquid solvent. In Fig. 1, we comparatively described the conventional heat-treatment and the liquid immersion heat-treatment for thermal crosslinking.

Young’s equation describes the surface energy difference of a solid substrate between air and liquid environments;


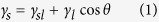


where *γ*_*s*_ and *γ*_*sl*_ are the surface energy of the solid substrate in a pure state and the wetting state by a liquid, respectively, *γ*_*l*_ is the liquid surface energy, and *θ* is the contact angle^20^. For a partial to complete wetting state, the term *γ*_*l*_ *cos θ* is always positive, and thus *γ*_*sl*_ is smaller than *γ*_*s*_. This implies that the surface energy of solid substrates can be lowered as long as they can be wetted by a liquid; increased wetting or wetting by a high-surface-energy liquid can greatly lower the solid surface energy. In other words, immersion of a nanopattern in a liquid that wets the nanopattern surface relieves the surface energy of the nanopattern and subsequently may retard its collapse during thermal crosslinking. Meanwhile, other requirements for the immersion liquid are that it should not be compatible with SU8 polymers; otherwise the liquid can swell or dissolve the polymer, which will thereby allow for easy flowing upon heating. Moreover, the liquid should have a high enough boiling point (b.p.) that the thermal crosslinking can be conducted at high temperature.

Here, we chose hexadecane as the immersion liquid. Hexadecane is one of a few high-b.p. liquids (290 °C). Hexadecane completely wetted the SU8 surface, as shown in Figure S2a. Hexadecane is a non-polar solvent and may thus be non-compatible with the SU8. A more accurate estimation of compatibility was obtained by evaluating the relative energy difference (*RED*) number based on the Hansen solubility parameter (25 °C), as shown in Table S1^21^. A *RED* value less than 1.0 indicates high affinity and a higher *RED* (i.e., >1.0) indicates lower affinity. Hexadecane clearly showed non-compatibility with the SU8. We tested a similar high-b.p. solvent, triethylene glycol (b.p., 285 °C), for comparison. Triethylene glycol partially wetted the SU8, as shown in Figure S2b. However, triethylene glycol is a high-polarity liquid. Based on its *RED* number, triethylene glycol was more compatible with the SU8 compared with hexadecane.

Figure 2b,c display the SEM images of 3D SU8 nanopatterns after heat treatment at 200 °C in the immersion liquid of hexadecane and triethylene glycol, respectively. Figure 2b shows that, in contrast to the sample heated in air (Figure S1), the wood-pile pattern maintained the porous structure, as expected. Specifically, the changes in the line width, line-to-line distance, and layer-by-layer distance of the wood-pile structure were less than 5%. By contrast, Fig. 2c displays a partially melted and resultant pore-collapsed morphology. Specifically, although the *RED* number of triethylene glycol was >1.0 for the SU8, the solubility parameters decreased with increasing temperature; in particular, the hydrogen bonding parameter was most sensitive to the temperature because hydrogen bonding was more likely to be broken^22^. Thus, triethylene glycol may swell the SU8 nanopatterns at high temperature, which induced the pattern melting.

**Figure 3:**
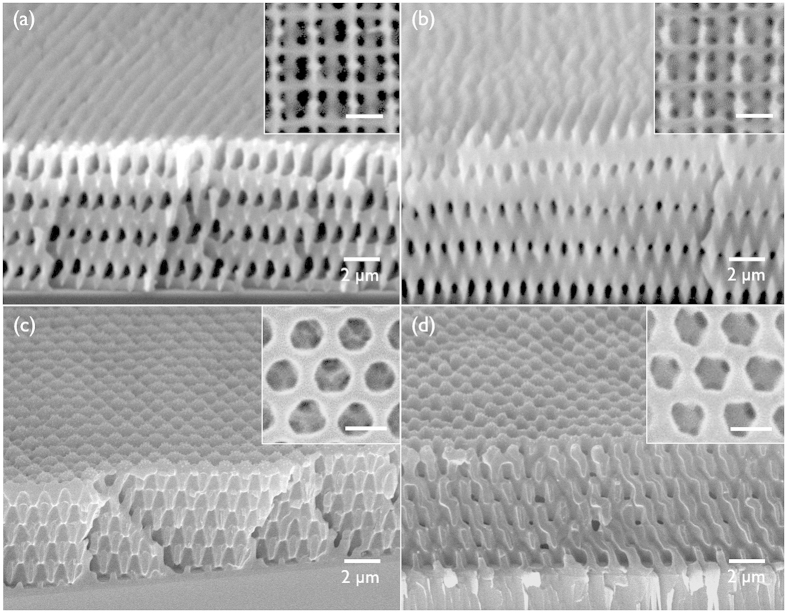
(**a**,**c**) SEM images of thin-skeleton wood-pile pattern and *FCC* pattern in a tilted view, respectively. (**b**,**d**) SEM images of each pattern treated at 200 °C immersed in hexadecane. The insets show the surface image of each pattern. (Scale bar, 1 μm).

We tested the liquid immersion heat-treatment on different types of 3D SU8 nanopatterns; the same wood-pile pattern but with thin skeleton (approximately 150 nm line width) prepared by oxygen plasma etching (1400 Torr, 15 min) (Fig. 3a) of the as-prepared pattern, and a face-centred-cubic (*FCC*)-like symmetry pattern prepared using four-beam interference pattern (Fig. 3c). Figure 3b,d display the SEM images of each pattern after heat treatment at 200 °C in the immersion liquid of hexadecane. The pattern structure and dimension are maintained during the heat-treatment. Thus, the liquid immersion heat-treatment is effective to various morphologies and pattern dimension.

**Figure 4:**
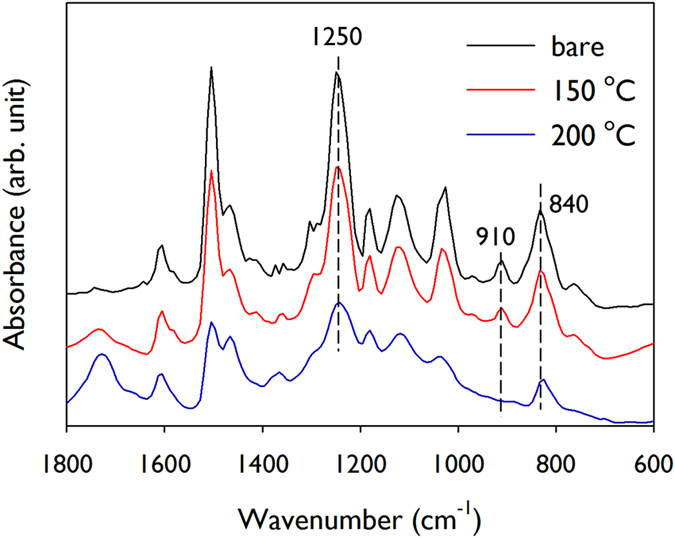
FTIR spectra of SU8 films with and without liquid immersion heat treatment at 150 °C and 200 °C.

The crosslinking reaction during the hexadecane immersion heat treatment was characterised using FTIR (see the reaction in Figure S3). Figure 4 displays the FTIR spectra of SU8 films with and without the heat treatment. The spectra were normalised to the peak of the aromatic ring at 1608 cm^−1^ because this group constitutes the backbone of the SU8 monomers. The peaks at 910 cm^−1^, 840 cm^−1^, and 1250 cm^−1^ were categorized as the epoxide group^18^. In the SU8 molecules, the peaks at 840 cm^−1^ and 1250 cm^−1^ overlapped with the para-substituted aromatic ring and aromatic ether band, respectively^23^. Thus, the peak at 910 cm^−1^ can be considered the characteristic peak of the epoxide group in the SU8^24^. In Fig. 4, the peak at 910 cm^−1^ was not observed in the spectrum after the hexadecane immersion heat treatment. Both the 840 and 1250 cm^−1^ peaks also decreased during the heat treatment. These results confirm the ring opening of the epoxide group that may be induced by the crosslinking reaction. The aliphatic ether peak (1050–1150 cm^−1^) produced by the epoxy crosslinking was not remarkable; this may be because these peaks overlapped with the C-H in-plane bending of the aromatic rings (1020–1200 cm^−1^)^23^. Meanwhile, the carbonyl peak at 1730 cm^−1^ appeared, whereas the aromatic ether peak (1250 cm^−1^) and aliphatic ether peaks (1050–1150 cm^−1^) decreased. This implies that some ether groups decomposed into the carbonyl group during the heat treatment^18^. The polymer crosslinking could also be directly proven by the enhancement of mechanical strength^25^. The elastic modulus and the hardness of the SU8 nanopatterns were measured via nanoindentation, as shown in Figure S4. Both the elastic modulus and hardness were largely increased with the liquid immersion baking temperature.

### Carbonisation of 3D nanopatterns and characterisation of the carbon patterns

**Figure 5:**
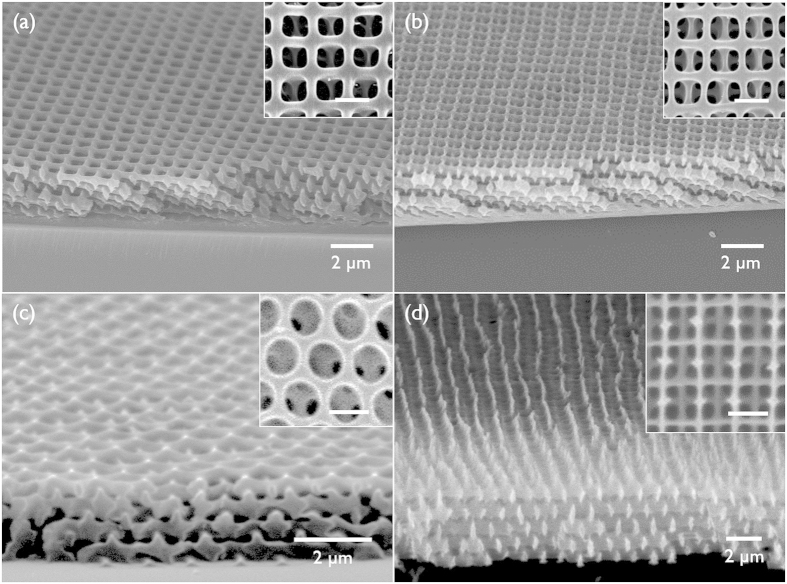
(**a**,**b**) SEM images of wood-pile carbon patterns prepared at 700 °C and 900 °C, respectively. (**c**,**d**) SEM images of *FCC* and thin-skeleton carbon patterns, respectively. The insets show the surface image of each pattern. (Scale bar, 1 μm).

The carbonisation of 3D SU8 nanopatterns heat-treated by liquid immersion was achieved via heat treatment 700 °C and 900 °C under an argon atmosphere. Figure 5a,b are SEM images of the carbonised wood-pile patterns at 700°C and 900 °C, respectively. The porous structures in the patterns were clearly maintained during the carbonisation. However, the wood-pile carbon patterns displayed shrinkage at both temperatures compared with the polymer patterns; the line width in the pattern shrank by 50% for 700 °C sample and 55% for 900 °C sample, respectively, the line-to-line distance on the surface revealed 5% shrinkage for both samples, and the layer-by-layer distance shrank by 50% for 700 °C sample and 55% for 900 °C sample. Figure S5 displays the thermogravimetric (TGA) analysis of the SU8 patterns up to 900 °C. The result displayed a mass loss of up to 25% of the initial weight at approximately 700–900 °C. A large shrinkage of the line width in the pattern was due to the pyrolytic carbonisation. The anisotropic shrinkage along the thickness dimension was induced by strong adhesion of the nanopattern onto the substrate. The surface area of these patterns was evaluated by the measurement of electrochemically active surface area. Briefly, the electrochemically active surface area was estimated by using the Randles-Sevcik approach.(see Figure S6) The active surface area of the wood-pile carbon patterns prepared at 700 °C and 900 °C were similar to each other. The surface area of the wood-pile carbon patterns are approximately 4 times higher than the carbon film prepared without the liquid immersion step.

Figure 5c,d display SEM images of the carbonised patterns of the *FCC* pattern and thin-skeleton wood-pile pattern. We observed the shrinkage in the width of skeleton by approximately 50%. Specifically, the thin-skeleton carbon pattern in Fig. 5d possesses only 80 nm thick skeleton while maintains high structural integrity. Moreover, we prepared a porous film with smaller diameter than the *FCC* or wood-pile patterns by using a colloidal crystal template. (see Figure S7) The pore size of colloidal crystal-templated film was approximately 200 nm, which was approximately 5 times smaller than the rectangular window size of wood-pile patterns. We applied the liquid immersion thermal crosslinking and subsequent carbonisation for this porous film. The SEM result clearly showed that the porous film successfully converted into the carbon film maintaining the porous structure. These results confirm that the liquid immersion heat treatment enhances high enough thermal resistivity of various 3D patterns during the pyrolytic carbonisation, although along with large mass loss.

**Figure 6:**
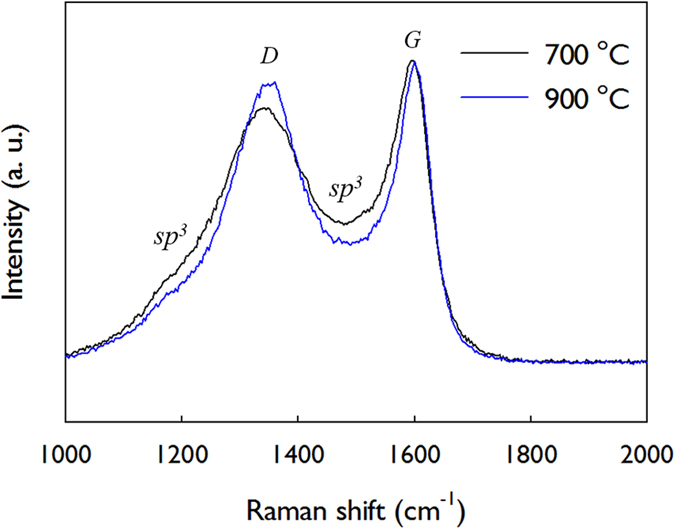
Raman spectra of 3D carbon patterns obtainned at 700 and 900 °C.

The SU8-derived carbon materials were characterised using Raman spectroscopy and XPS. Figure 6 shows the Raman spectra of carbon nanopatterns prepared at 700 and 900 °C. Two characteristic peaks appeared at approximately 1590 cm^−1^ and 1340 cm^−1^ under visible excitation from the carbonaceous materials; the former represented the *E*_*2g*_ symmetry mode (often labelled the *G* mode as in graphite) and the latter appeared from the *A*_*1g*_ symmetry of disordered graphite (designated as the *D* mode)^26^,^27^. Moreover, the spectra displays the shoulder peaks around 1180 cm^−1^ and 1500 cm^−1^, which have been assigned as the contribution from *sp*^3^ carbon^28^,^29^. The similar intensities of *D* and *G* peaks of the carbon patterns in Fig. 6 have been often observed in a polymer-derived carbon that possess microcrystalline graphite characteristics^26^,^27^. Specifically, as the carbonisation temperature increased, the shoulder peaks are reduced while the D peak is increased. This result indicates that more graphitic domains are created by the conversion of amorphous *sp*^*3*^ carbon at higher temperature.

**Figure 7:**
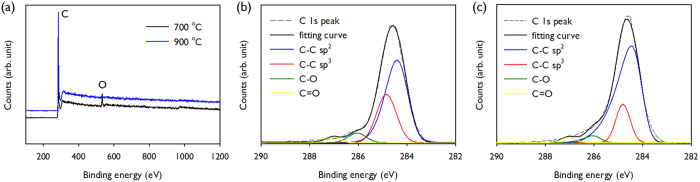
(**a**) XPS survey spectra and (**b**,**c**) C1s spectra of 3D carbon patterns obtained at 700 and 900 °C. The spectra were deconvoluted into four spectra of *sp*^*2*^ C-C, *sp*^*3*^ C-C, C-O and C=O functionalities.

Figure 7a displays the wide-scan XPS spectrum of the carbon patterns prepared at 700 °C and 900 °C. The oxygen content for each sample was approximately 3 at. %. Because the atomic percent of oxygen in the SU8 was measured at approximately 18 at. % higher than the carbon content, most of the oxygenated groups were removed during carbonisation. Figure 7b,c show C 1s peaks from the carbon nanopatterns prepared at 700 °C and 900 °C, respectively. The peak was deconvoluted into four peaks at different binding energies: *sp*^*2*^ C-C bonding at 284.3–284.6 eV, *sp*^*3*^ C-C bonding at 284.8–285.1 eV, C-O group at 285.5–286.0 eV and C=O group at 287.0–288.1 eV^30^,^31^. The *sp*^*2*^ peak was fitted by an asymmetric Lorentzian function, and the others were fitted by Gaussian peaks. Table S2 displays the summary of the atomic percent range of these bonds. Comparing the area ratios of the *sp*^*2*^ and *sp*^*3*^ atomic contents, the carbon patterns prepared at higher temperature exhibited more *sp*^*2*^ content than *sp*^*3*^, which was consistent with the Raman analysis. We measured the electrical conductivity of the wood-pile carbon patterns by 4-point probe analysis. The conductivity value of the carbon patterns prepared at 900 °C was approximately 3.9 × 10^3^ S/m, which was several orders of magnitude higher than the carbon prepared at 700 °C. The high conductivity for 900 °C carbon patterns may be attributed to high content of *sp*^*2*^ bonding.

**Figure 8:**
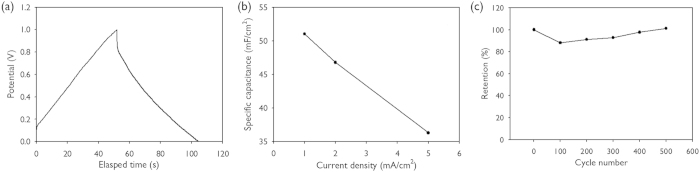
(**a**) Galvanostatic charging/discharging curves of wood-pile carbon pattern electrodes at a current density of 1 mA/cm^2^. (**b**) Specific capacitances at various current densities (**c**) Capacitance retention over charging/discharging cycles at a current density of 1 mA/cm^2^.

Finally, we applied the wood-pile carbon nanopattern as an electrode for supercapacitors. Specifically, the nitrogen-doped carbon nanopattern was prepared similar to that of our previous report^14^. The galvanostatic charging/discharging curve of wood-pile carbon pattern electrodes is shown in Fig. 8a. The specific capacitance was estimated to be 52 mF/cm^2^ at a current density of 1 mA/cm^2^ (Fig. 8), which was 63% higher than that of previous result. In contrast to the previous approach, where the doping was achieved through the silica layer, the direct doping of nitrogen into carbon matrix in the liquid immersion-assisted carbonisation enhances the doping content, resulting in higher specific capacitance. The specific capacitance at various current densities is shown in Fig. 8b. Compared to the capacitance at 1 mA/cm^2^, the specific capacitance at a 5 times higher current density is 37 mF/cm^2^. The cycle performance was evaluated by the measurement of capacitance over charging/discharging cycles as shown in Fig. 8c. The capacitance retention was maintained within 90% of the initial capacitance during 500 cycles, which demonstrates the good cycle stability of the carbon nanopattern film as a supercapacitor electrode.

## Conclusion

We have demonstrated a novel and facile liquid immersion heat treatment strategy to crosslink polymer nanopatterns without inducing any pattern morphology change. The strategy is based on the relief of nanopatterns’ high surface energy by wetting the surface with a liquid. On the basis of high b.p., non-compatibility with the polymer, and wetting behaviour, we showed that hexadecane was a good candidate for this purpose. In contrast to the pore collapse of 3D porous SU8 nanopatterns during heat treatment in an air environment, thermal crosslinking immersed in hexadecane clearly showed no such issue. The FTIR and the mechanical modulus measurement clearly indicated the crosslinking of SU8 epoxide groups during the liquid immersion heat treatment. Moreover, we observed that the liquid immersion thermal crosslinked 3D patterns were converted into carbon nanopatterns with high structural integrity; the skeleton of the nanopattern became thin because the pyrolytic decomposition during the carbonisation. The Raman and XPS analysis revealed that a large portion of *sp*^*2*^ carbon configuration was produced during the high temperature carbonisation. We applied the carbon nanopattern as an electrode for supercapacitors. The galvanostatic charging/discharging analysis revealed the specific capacitance of 52 mF/cm^2^ at a current density of 1 mA/cm^2^, specifically which was 63% higher than that of previous result^14^. We believe that this facile liquid immersion technique to control interfacial energy can be used for the heat treatment of a variety of polymer nanopatterns with structural integrity. It can also be employed in processes such as thermal self-assembly and annealing of block copolymer patterning and hard-baking of MEMS patterns.

## Additional Information

**How to cite this article**: Kang, D.-Y. *et al.* Liquid immersion thermal crosslinking of 3D polymer nanopatterns for direct carbonisation with high structural integrity. *Sci. Rep.* **5**, 18185; doi: 10.1038/srep18185 (2015).

## Supplementary Material

Supplementary Information
