# Supplementary material for: Liquid immersion thermal crosslinking of 3D polymer nanopatterns for direct carbonisation with high structural integrity
Source: Sci Rep. 2015 Dec 18;5:18185. doi: 10.1038/srep18185 (PMC4683537; doi:10.1038/srep18185)
Supplement: Supplementary Information [file srep18185-s1.doc]

Supporting Information

Liquid immersion thermal crosslinking of
3D polymer nanopatterns for direct carbonisation with high structural integrity

Da-Young Kang, Cheolho Kim, Gyurim Park and Jun Hyuk Moon*

Department of Chemical and Biomolecular Engineering, Sogang University, Seoul 121-742, South Korea

Corresponding author, E-mail: [junhyuk@sogang.ac.kr](mailto:junhyuk@sogang.ac.kr)


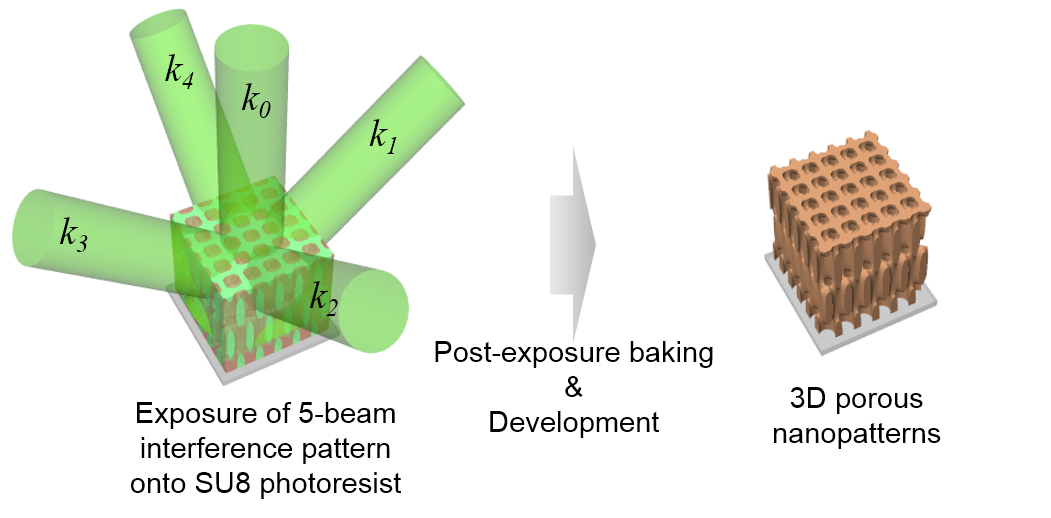


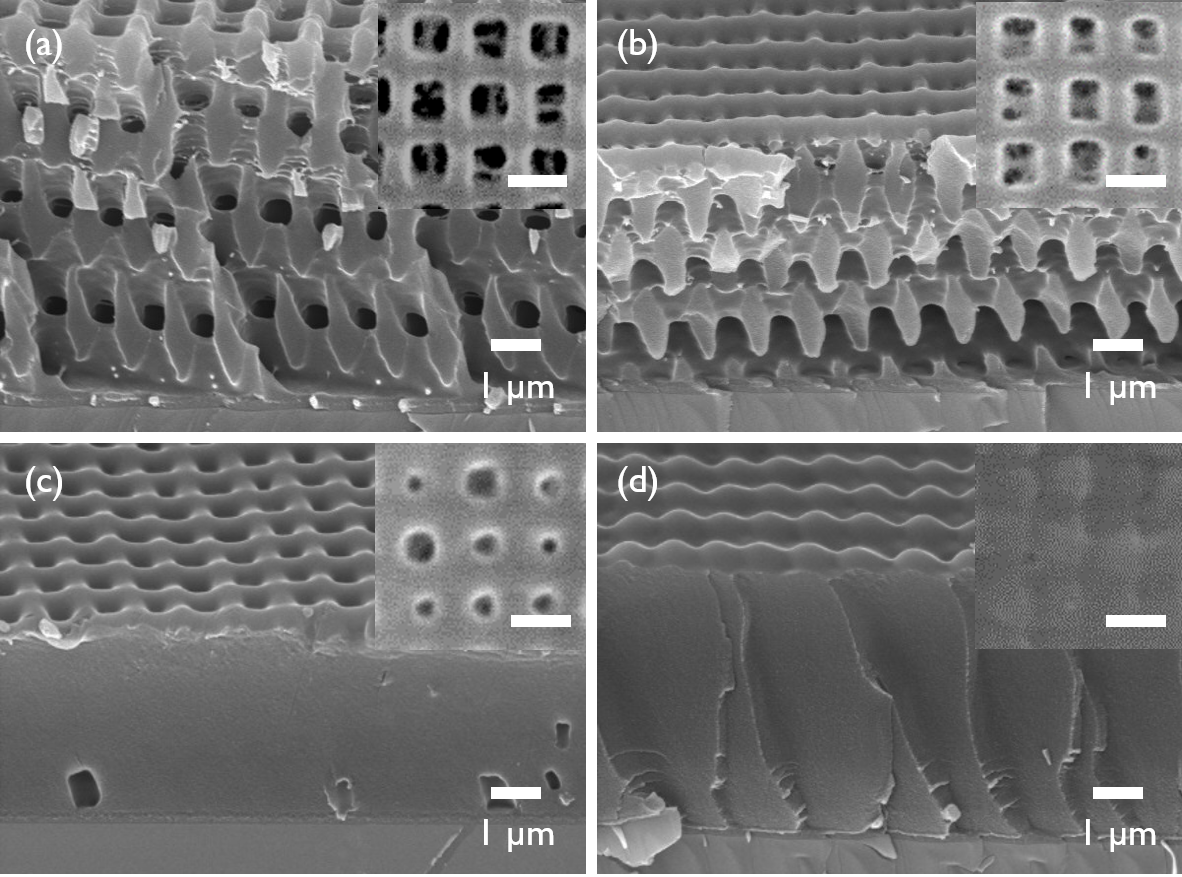


Figure S1. (up) Multi-beam laser interference lithography for fabricating 3D wood-pile nanopatterns. (down) Cross-sectional SEM images of SU8 photoresist 3D patterns (a) without and (b,c,d) with heat treatment at 100°C, 120°C, and 150°C, respectively.


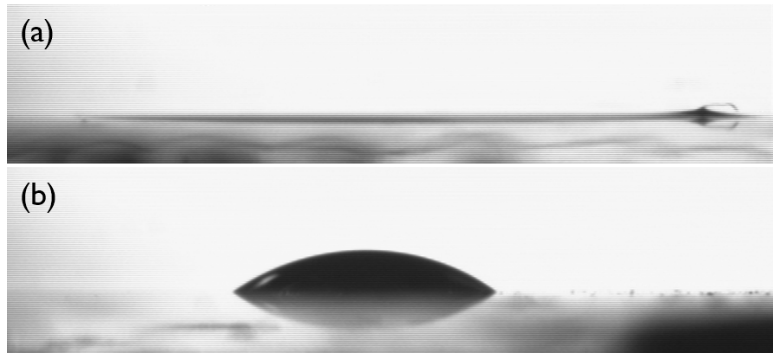


Figure S2. Digital camera image of (a) a hexadecane droplet on an SU8 film and (b) a triethylene glycol droplet on an SU8 film.

Table S1. Hansen solubility parameters of the SU8, hexadecane, and triethylene glycol. *δ_d_*, *δ_p_* and *δ_h_* are the dispersion, polarity and hydrogen bonding energies, respectively. *R_0_* is the interaction radius. *R_a_* is the distance between the Hansen parameters of the solvent and SU8. The relative energy difference (RED) is the ratio of *R_a_* to *R_0_*.

|  | *δ_d_* | *δ_p_* | *δ_h_* | *R_0_* | *R_a_* | RED |
| --- | --- | --- | --- | --- | --- | --- |
| SU8 | 18.1 | 11.4 | 9 | 9.1 |  |  |
| Hexadecane | 16.3 | 0 | 0 |  | 14.96 | 1.6 |
| triethylene glycol | 16 | 12.5 | 18.6 |  | 10.53 | 1.1 |


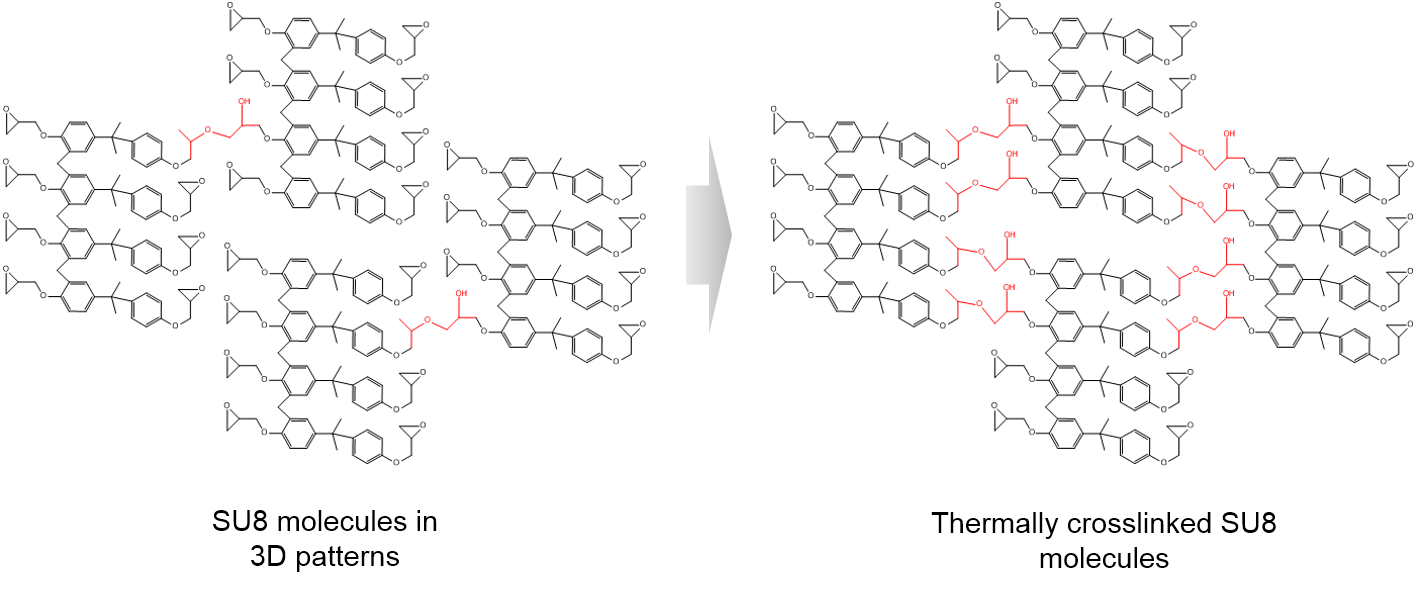


Figure S3. Crosslinking reaction of SU8 molecules; (left) SU8 patterns, weakly crosslinked. (right) fully crosslinked SU8 molecules by the liquid immersion thermal crosslinking. The crosslinked bond is coloured in red.


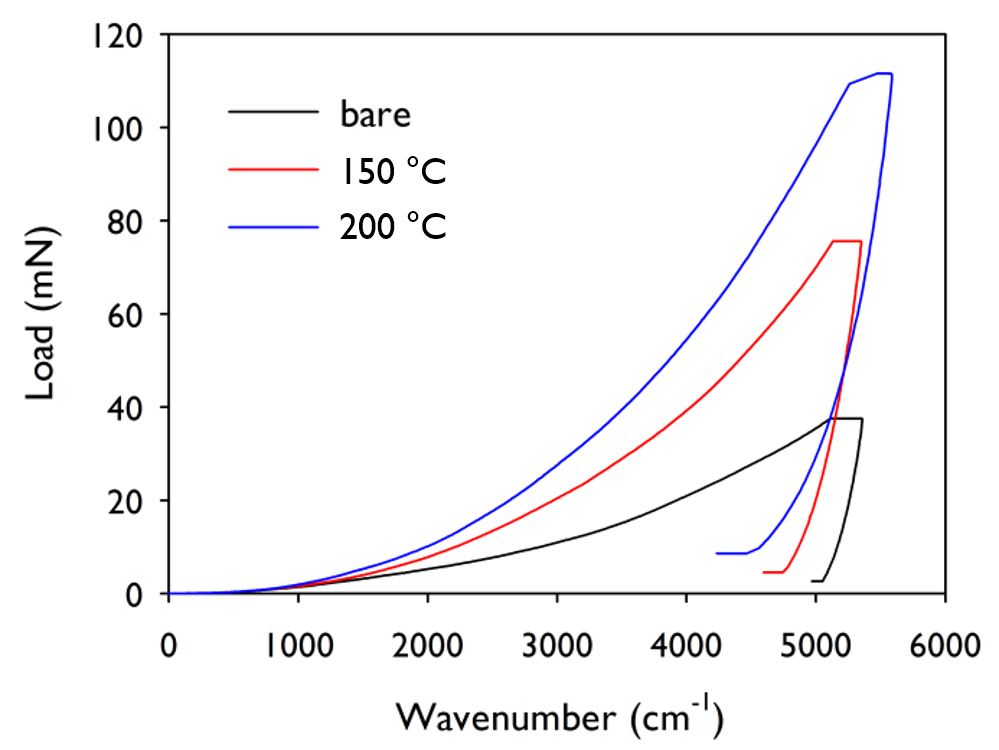


Figure S4. Nanoindentation curves of 3D patterns with and without liquid immersion heat treatment at 150°C and 200°C. The elastic modulus (E) and the hardness (H) were estimated based on the load-displacement results. The E was calculated from the unload curve by using the Hertzian theory. The H was approximated as the indentation load divided by the projected contact area of the indentation. The E (GPa), H (GPa) values for bare, 150°C, and 200°C were (1.94, 0.06), (2.47, 0.08), (2.74, 0.11), respectively.


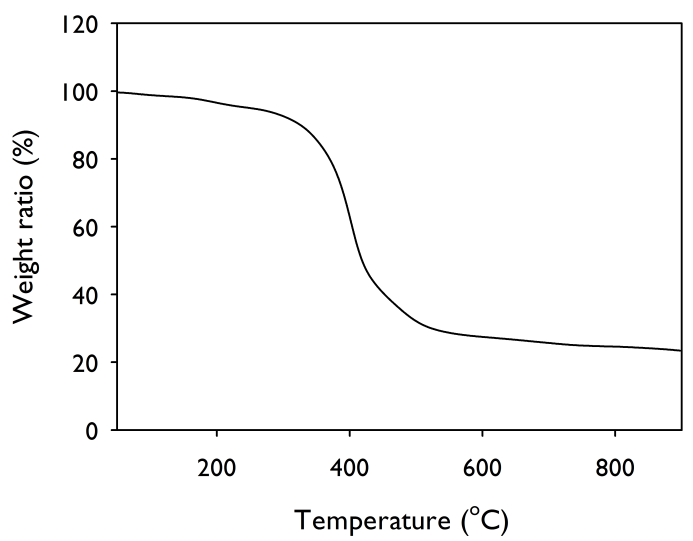


Figure S5. TGA of SU8 treated at 200°C.


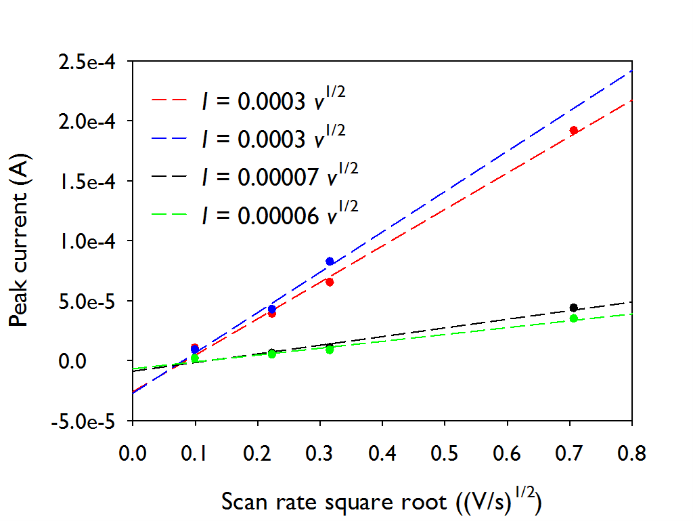


Figure S6. The relationship between the peak current and the square root of scan rate fitted by the Randles-Sevcik equation for the wood-pile carbon patterns prepared at 700 ºC (blue), and at 900 ºC (red). The non-patterned carbon film (black) and the carbonized film without the liquid-immersion heat-treatment step (green) was prepared for comparison. Here, the electrochemical active surface area can be estimated by the Randles-Sevcik equation, *I_p_ =268,600 n^3/2^ A D^1/2^ C v^1/2^*, where *I_p_* is the peak current (A), *A* is the electroactive area (cm^2^), *C* is the concentration of the electroactive specie (mol cm^-3^), *n* is the number of exchanged electrons, *D* is the diffusion coefficient (cm^2^ s^-1^) and *v* is the scan rate (V/s). In the range of scan rate, the current varies linearly with the square root of the scan rate. Here, the slope of Randles-Sevcik plots is proportional to the active surface area.


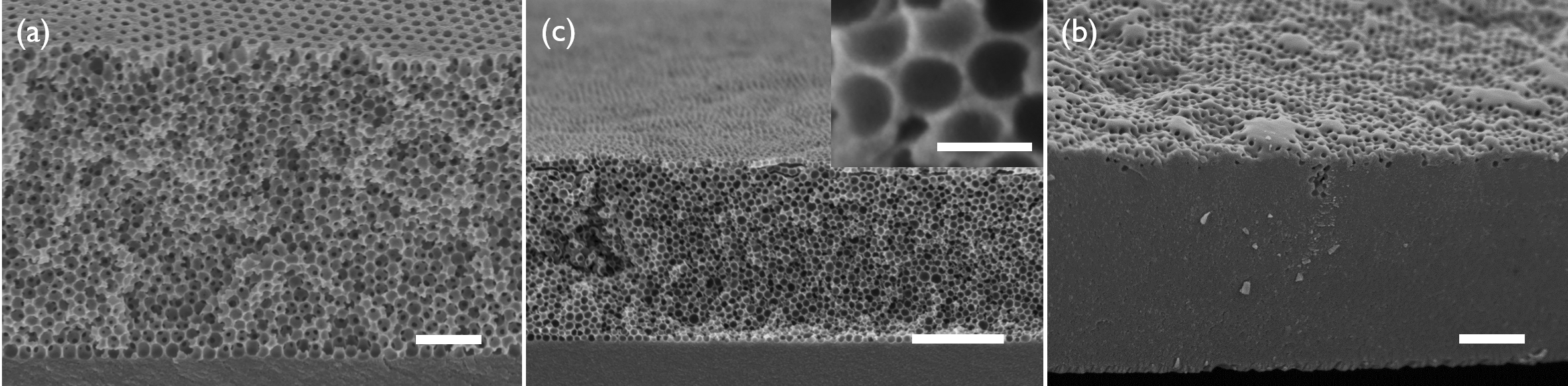


Figure S7. (a) Cross-sectional SEM image of SU8 porous film prepared using colloidal crystal templates. A carbonized film (b) with and (c) without the liquid immersion thermal treatment. (scale bar: 1 μm, scale bar of inset image: 100 nm) The porous film was prepared in a different approach using colloidal crystal templates. Briefly, we deposited monodispersed silica particle film, infiltrated SU8 resin, and then, removed the silica particle templates.

Table S2. Summary of the atomic percent of chemical bonds characterized by XPS measurement.

| Chemical bonding | Atomic % (at. %) | |
| --- | --- | --- |
|  | 700 °C | 900 °C |
| C-C, *sp^2^* | 54.3 – 64.4 | 65.4 – 70.9 |
| C-C, *sp^3^* | 24.9 – 36.3 | 20.1 – 26.1 |
| C-O | 6.6 – 7.8 | 4.8 – 6.2 |
| C=O | 1.6 – 3.9 | 3.6 – 3.7 |
